# Effects of Different Intensities and Durations of Aerobic Exercise on Vascular Endothelial Function in Middle-Aged and Elderly People: A Meta-analysis

**DOI:** 10.3389/fphys.2021.803102

**Published:** 2022-01-21

**Authors:** Qiuping You, Laikang Yu, Gen Li, Hui He, Yuanyuan Lv

**Affiliations:** ^1^Sports Coaching College, Beijing Sport University, Beijing, China; ^2^Department of Strength and Conditioning Training, Beijing Sport University, Beijing, China; ^3^China Institute of Sport and Health Science, Beijing Sport University, Beijing, China

**Keywords:** aerobic exercise, endothelial function, flow-mediated dilation, middle-aged people, elderly people

## Abstract

**Background:**

Previous studies have found that aerobic exercise was more effective in improving vascular endothelial function than resistance training, high-intensity interval training (HIIT), and other types of exercise, while the effects between different intensities and durations of aerobic exercise were unclear. Therefore, we performed this meta-analysis to investigate the effects of different intensities and durations of aerobic exercise on the vascular endothelial function of middle-aged and elderly people.

**Methods:**

Databases were searched up to April 2021 for studies evaluating the influences of different intensities and durations of aerobic exercise on endothelial function assessed by flow-mediated dilation (FMD) among healthy middle-aged and elderly people. Data were pooled using random-effects models to obtain the weighted mean difference (WMD) and 95% confidence intervals (CIs).

**Results:**

A total of 9 studies involving 221 participants fulfilled the inclusion criteria. Aerobic exercise improved the overall FMD of healthy middle-aged and elderly people [WMD, 1.33 (95% CI, 0.37–2.28), *P* < 0.05]. Specifically, vigorous-intensity exercise increased FMD significantly in healthy middle-aged and elderly people [WMD, 1.10 (95% CI, 0.27–1.93), *P* < 0.05], while moderate-intensity exercise had no significant association with FMD [WMD, 1.49 (95% CI, −0.62 to 3.60), *P* = 0.17]. In addition, long-term (8 weeks or above) aerobic exercise increased the FMD in healthy middle-aged and elderly people [WMD, 1.63 (95% CI, 0.61–2.66), *P* < 0.05], while one-time acute aerobic exercise had no significant association with FMD of healthy middle-aged and elderly people [WMD, 0.89 (95% CI, −1.47 to 3.24), *P* = 0.46]. Specifically, 8 weeks or above of vigorous-intensity exercise increased FMD significantly in healthy middle-aged and elderly people [WMD, 1.48 (95% CI, 1.06–1.90), *P* < 0.01], while 8 weeks or above of moderate aerobic exercise had no significant association with FMD [WMD, 1.49 (95% CI, −0.62 to 3.60), *P* = 0.17].

**Conclusion:**

Aerobic exercise, especially 8 weeks or above of vigorous-intensity aerobic exercise, improved the endothelial function in healthy middle-aged and elderly people.

## Introduction

Aging is an inevitable cardiovascular risk factor, and the increase in age will make the body more susceptible to pathological stress (Tian and Li, 2014) and increase the prevalence of various cardiovascular diseases (CVD) include arteriosclerosis, hypertension, stroke, and so on (North and Sinclair, 2012). Vascular endothelial cells are a single layer of cells adjacent to the lumen of blood vessels and play an important role in the regulation of vascular tone and the maintenance of hemodynamics (Jia et al., 2019), which can directly act on various cardiovascular and peripheral vascular diseases (Rajendran et al., 2013). Endothelium dysfunction, especially impaired endothelium-dependent vasodilation, has been linked to arterial stiffness, atherosclerosis, coronary artery disease, and so on (Ungvari et al., 2018). Flow-mediated dilation (FMD) of the brachial artery is currently used as a parameter in evaluating vasodilation (Thijssen et al., 2019) and is used to predict the risk of cardiovascular diseases in clinic studies, independently, which provides us with a non-invasive method to assess vascular endothelial function. In addition, the risk of CVD will increase 13% following every 1% reduction in brachial artery FMD (Inaba et al., 2010; Ras et al., 2013; Xu et al., 2014; Matsuzawa et al., 2015; Thijssen et al., 2019).

It is well-known that endothelial function is age-dependent (Ungvari et al., 2018). The epidemiologic study has shown that regular exercise can prevent CVD and reduce cardiovascular morbidity and mortality in the general population, especially healthy subjects (Eckel et al., 2014; Arnett et al., 2019; Seals et al., 2019). Therefore, this study pays more attention to the impact of aerobic exercise on improving vascular endothelial function in middle-aged and elderly people. Preliminary research suggested that aerobic exercise had a better effect than other types of exercise in improving vascular endothelial function and reducing the risk of CVD (Zhao et al., 2014; Boeno et al., 2020). The main mechanism was that aerobic exercise can improve the bioavailability of nitric oxide and reduce oxidative stress (Seals et al., 2019). However, some studies found that FMD does not always increase with the continuous training of 8–12 weeks (Tinken et al., 2010; Birk et al., 2012; Green et al., 2014; Green and Smith, 2018), which prompted that time characteristic was a key role. In addition, different aerobic exercise intensities have different effects on vascular endothelial function (Yoo et al., 2017; Green and Smith, 2018). In conclusion, there was not a unanimous result on the influence of different intensities and different durations of aerobic exercise on vascular endothelial function (Goto et al., 2003; Man et al., 2020). Therefore, we performed this meta-analysis to explore the effects of different intensities and durations of aerobic exercise on vascular endothelial function in healthy middle-aged and elderly people.

## Methods

### Design

This meta-analysis was reported following the Preferred Reporting Items for Systematic Reviews and Meta-Analyses (PRISMA) guidelines (Moher et al., 2015).

### Search Strategy

All the studies before April 2021 on aerobic exercise to improve the vascular endothelial function in middle-aged and elderly people were searched in PubMed and Web of Science, using the following MESH terms and text words: aerobic exercise, middle-aged, elderly people, and vascular endothelial function. We also hand-searched reference lists of all identified studies. All studies used for meta-analysis need to meet the following criteria: (1) the participants were healthy middle-aged and elderly people; (2) the intervention used in the study was aerobic exercise; (3)FMD was used for evaluating vascular endothelial function. Articles were excluded if the language was non-English or using an animal model. Reviews and conference articles were also excluded from the analysis. As Li et al. (2021) reported, middle-aged and elderly people was defined as people ≥45 years old.

### Data Extraction and Quality Assessment

The documental information of all qualified studies includes author information, participant characteristics (including sex distribution), age, training type, training intervention duration, training intensity, training frequency, time of one training, and research result indicators (FMD). Two reviewers (QY and LY) independently reviewed the titles, abstracts, and full texts of all citations to identify studies reporting the effects of aerobic exercise on vascular endothelial function in healthy middle-aged and elderly people. When the data could not be extracted or there was a dispute, two authors negotiated or contacted the author of the article to resolve it. Otherwise, the platform was used to extract the information (WebPlotDigitizer., 2021).

The Cochrane collaboration bias tool, which includes items on selection bias, performance bias, detection bias, attrition bias, and reporting bias, was used to evaluate the quality of eligible studies.

### Data Synthesis and Analysis

Data were pooled using random-effects models to obtain the weighted mean difference (WMD) and 95% confidence intervals (CIs). When analyzing whether aerobic exercise could improve the vascular endothelial function of healthy elderly people, the Chi-square (χ^2^) test was used. If there was a high level of heterogeneity in the test (*I*^2^ > 60%), we used subgroup analysis or sensitivity analysis to explain the results (Moher et al., 2015; Shamseer et al., 2015). In the subgroup analysis, we tried to use different intensities and durations of aerobic exercise to explore the impact on vascular endothelial function. The analysis result, funnel plot, and forest chart were generated using the software RevMan.5. In terms of overall impact, *P* < 0.05 was considered statistically significant.

## Results

### Studies Retrieved and Characteristics

**Figure 1:**
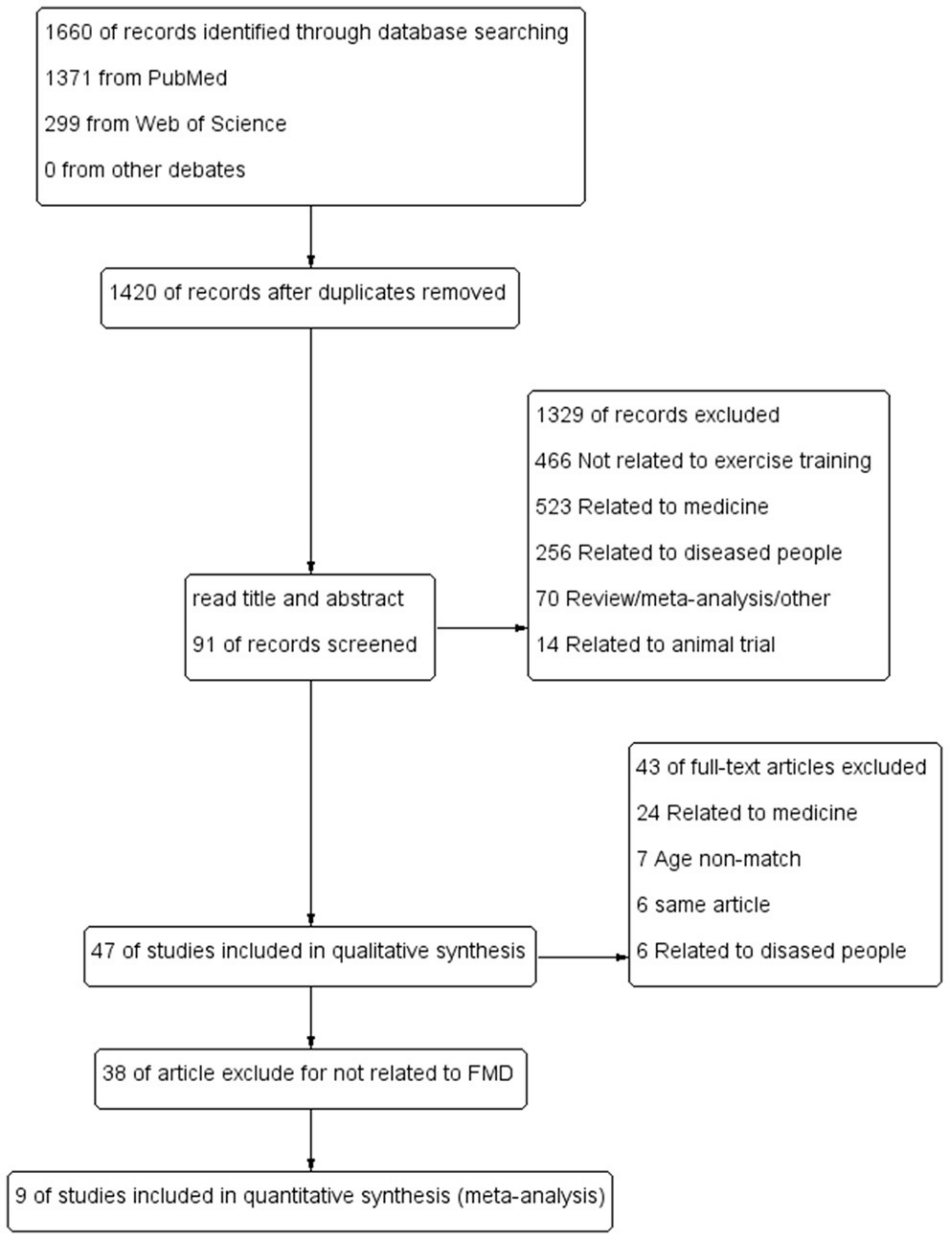
PRISMA flowchart of study selection.

The literature search results and research selection process were shown in Figure 1. Among the 1,426 articles identified, after reading the titles and abstracts, and then reading the full texts, 9 studies were considered eligible for meta-analysis (Nishiwaki et al., 2011; Pierce et al., 2011; Schaun et al., 2011; Akazawa et al., 2012; Swift et al., 2014; Serviente et al., 2016; Hunter et al., 2018; Bouaziz et al., 2019; Klonizakis et al., 2020).

The main characteristics of participants and exercise interventions were shown in Table 1. Nine studies involved 221 participants, of which 5 studies directly explored the effects of aerobic exercise on vascular endothelial function (Schaun et al., 2011; Akazawa et al., 2012; Swift et al., 2014; Serviente et al., 2016; Bouaziz et al., 2019), 4 studies explored factors related to aerobic exercise and endothelial function (Nishiwaki et al., 2011; Pierce et al., 2011; Schaun et al., 2011; Serviente et al., 2016), these articles contained perimenopausal women and postmenopausal women (Nishiwaki et al., 2011; Pierce et al., 2011; Akazawa et al., 2012; Swift et al., 2014; Serviente et al., 2016; Hunter et al., 2018; Bouaziz et al., 2019; Klonizakis et al., 2020), only one article discussed all men (Schaun et al., 2011). According to the position statement of physical activity and training intensity (Norton et al., 2010), we adjusted the intensity classification of aerobic exercise according to the included research situation: 1.6 < METs <3, 20% < maximal oxygen uptake (VO_2max_) <40%, 40% < maximal heart rate (HR_max_) <55%, or 8 < RPE <10 were determined as light-intensity; 3 < METs <6, 40% < VO_2max_ <60%, 55% < HR_max_ <70%, or 11 < RPE <13 were determined as moderate-intensity; 6 < METs <9, 60% < VO_2max_ <85%, 70% < HR_max_ <90%, or 14 < RPE <16 were determined as vigorous intensity.

**Table 1:** Characteristics of studies included in this meta-analysis.

**Included studies**	**Sample**	**Age**	**Type**	**Intensity**	**Duration**	**Frequency**	**Times**	**Results**	**Conclusion**
Nishiwaki et al. (2011)	Normoxic group (*n* = 8); hypoxic group (*n* = 8)	Total 56 ± 1	AE	50% VO_2max_	8 weeks	4 days/week	30 min/day	FMD	+
Pierce et al. (2011)	Intervention study (*n* = 44); exercise (*n* = 22)	63 ± 1	AE	70–75% HR_max_	8 weeks	6–7 days/week	40–50 min/day	FMD	+
Schaun et al. (2011)	Male volunteers (*n* = 20)	54 ± 4	AE	65% HR_max_	12 weeks	3 days/week	30 min/day	FMD	+
Serviente et al. (2016)	Menopausal women (*n* = 15)	58.9 ± 1.4	AE	60–64% VO_2max_	30 min	one time	30 min/day	FMD	+
Hunter et al. (2018)	23 °C yoga (*n* = 14); 40.5 °C yoga (*n* = 19)	23 °C Yoga 49 ± 5; 40.5 °C Yoga 47 ± 5	AE	Moderate-intensity	12 weeks	3 days/week	90 min/day	FMD	+
Bouaziz et al. (2019)	Sedentary volunteers (*n* = 30)	79 ± 2.5	AE	88% HR_max_	9.5 weeks	2 days/week	30 min/day	FMD	+
Klonizakis et al. (2020)	Aqua (*n* = 20); land (*n* = 20); mixed (*n* = 20)	Aqua: 63.7 ± 7; land: 65 ± 6; mixed: 66 ± 6	AE	3 METs	≥ 6 moths	≥ 2 days/week	60 min/day	FMD	+
Akazawa et al. (2012)	Postmenopausal women (*n* = 11)	59 ± 5	AE	60–75% HR_max_	8 weeks	3 days/week	30–60 min/day	FMD	+
Swift et al. (2014)	African American (*n* = 8); Caucasian (*n* = 16)	55.8 ± 1.7	AE	RPE: 10–12; RPE: 15-17	12 weeks	4 days/week	Unclear	FMD	+

*AE, aerobic exercise; FMD, flow-mediated dilation; VO_2max_, maximal oxygen consumption; HR_max_, maximum heart rate; MET, metabolic equivalent; RPE, rating of perceived exertion; “+” represents a positive result*.

### Risk of Bias

**Figure 2:**
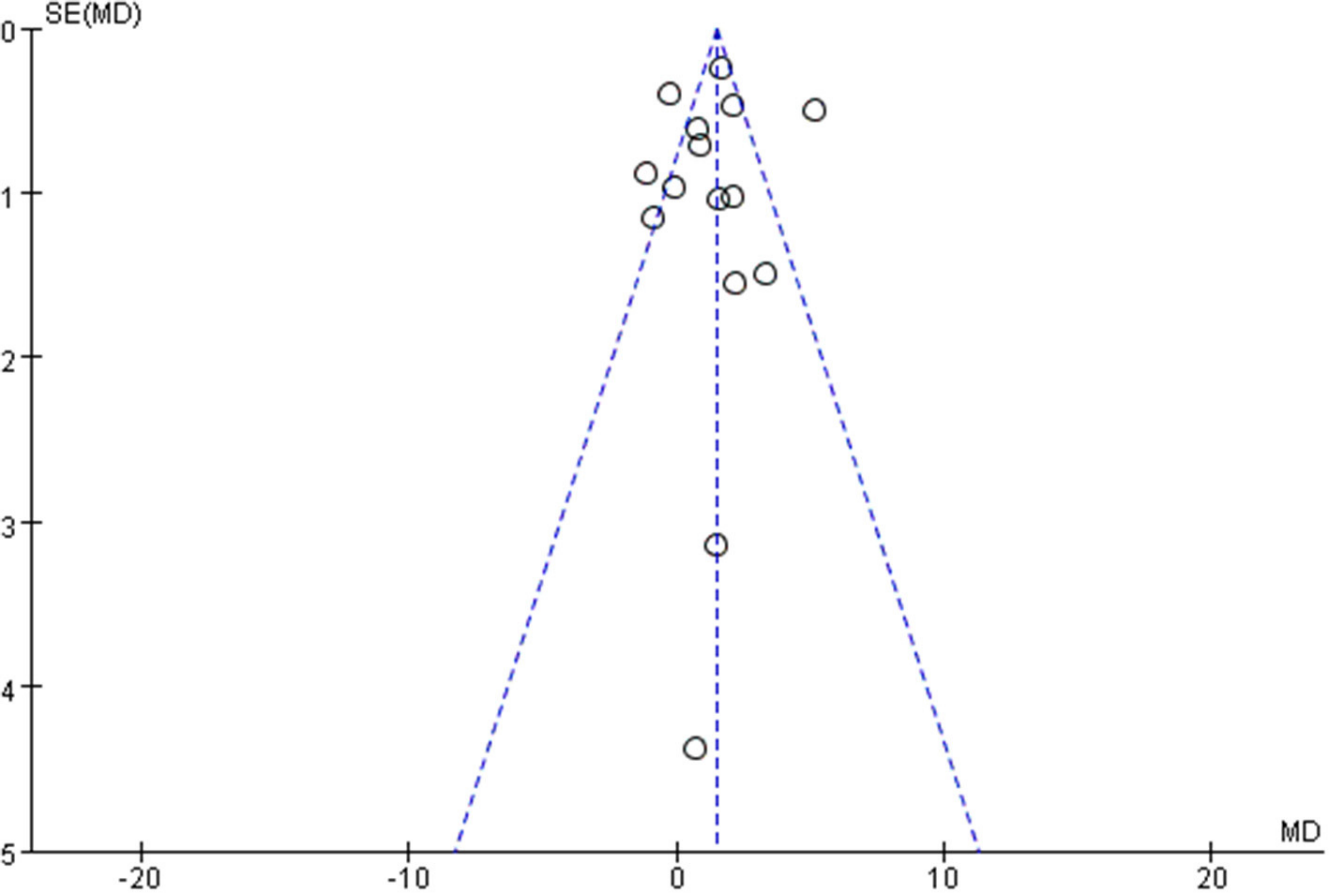
Funnel plot.

**Figure 3:**
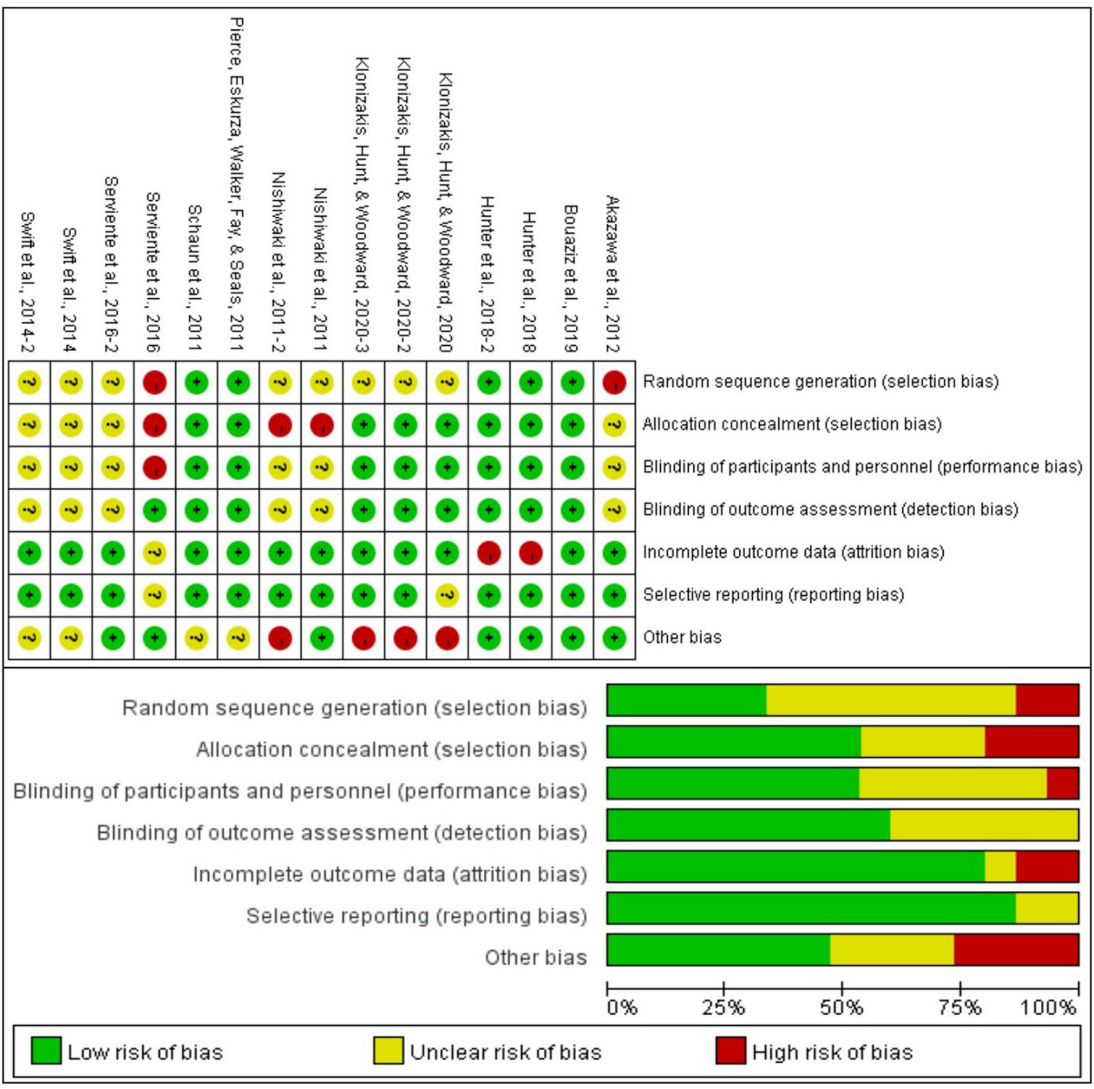
Results of Cochrane risk of bias tool.

Cochrane risk assessment tool was used to evaluate the methodological quality of the included literature, mainly from six aspects: selection bias, performance bias, detection bias, attrition bias, reporting bias, and other bias (Figure 2). The quality score was made according to three levels (low risk, high risk, and unclear). The quality of the included literature was divided into three levels from high to low: high quality, medium quality, and low quality (Figure 2). Publication bias was assessed visually by inspecting the funnel plot (Figure 3).

### Effects of Aerobic Exercise on the FMD

**Figure 4:**
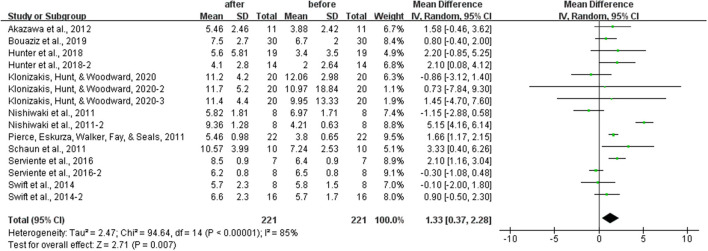
Meta-analysis results of the effects of aerobic exercise on the FMD in healthy middle-aged and elderly people. The pooled estimates were obtained from random effects analysis. Diamonds indicated the effect size of each study summarized as WMD. The size of the shaded squares was proportional to the percentage weight of each study. Horizontal lines represented the 95% CI and the vertical line represented the overall effect. Aerobic exercise improved FMD significantly [WMD, 1.33 (95% CI, 0.37–2.28), *P* < 0.05].

After analyzing the data of all included studies, we found that aerobic exercise could increase the FMD of healthy middle-aged and elderly people. However, this increase did not distinguish between exercise intensities and durations. As shown in Figure 4, aerobic exercise increased the FMD significantly [WMD, 1.33 (95% CI, 0.37–2.28), *P* < 0.05], while there was a significant heterogeneity (*I*^2^ = 85%, Figure 4).

### Subgroup Analysis: Effects of Different Intensities of Aerobic Exercise on the FMD

**Figure 5:**
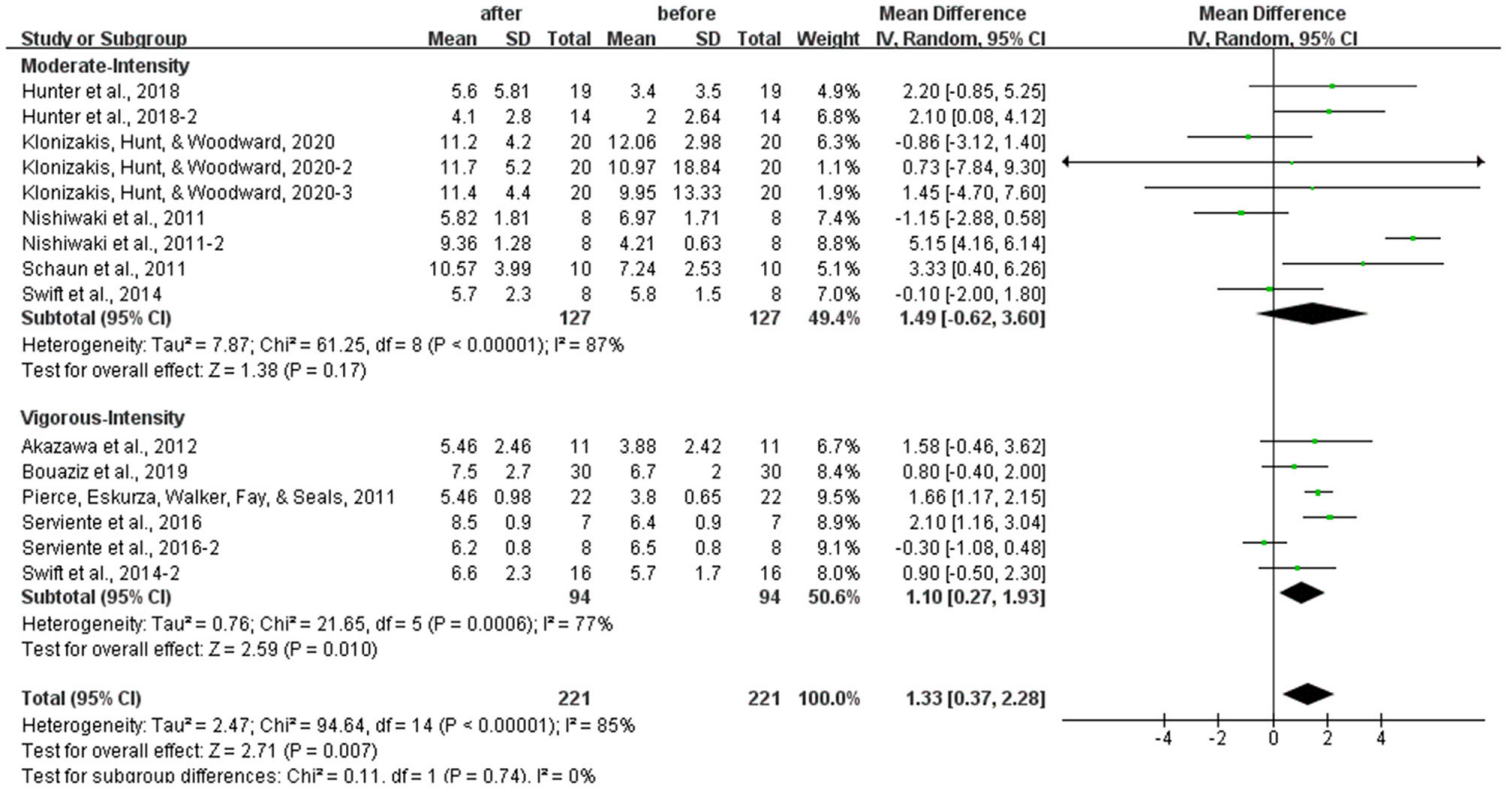
Meta-analysis of the effects of different intensities of aerobic exercise on the FMD in healthy middle-aged and elderly people. The pooled estimates were obtained from random effects analysis. Diamonds indicated the effect size of each study summarized as WMD. The size of the shaded squares was proportional to the percentage weight of each study. Horizontal lines represented the 95% CI and the vertical line represented the overall effect. Moderate-intensity aerobic exercise had no significant relationship with FMD in the middle-aged and elderly people [WMD, 1.49 (95% CI, −0.62 to 3.60), *P* = 0.17], while vigorous-intensity aerobic exercise improved FMD significantly [WMD, 1.10 (95% CI, 0.27–1.93), *P* < 0.05].

Different results were shown when considering exercises intensities. Specifically, vigorous-intensity exercise increased the FMD significantly [WMD, 1.10 (95% CI, 0.27–1.93), *P* < 0.05], while moderate-intensity exercise had no significant association with FMD in healthy middle-aged and elderly people [WMD, 1.49 (95% CI, −0.62 to 3.60), *P* = 0.17]. However, both subgroups had significant heterogeneity (*I*^2^ = 77% and *I*^2^ = 87%, respectively; Figure 5).

### Subgroup Analysis: Effects of Different Durations of Aerobic Exercise on the FMD

**Figure 6:**
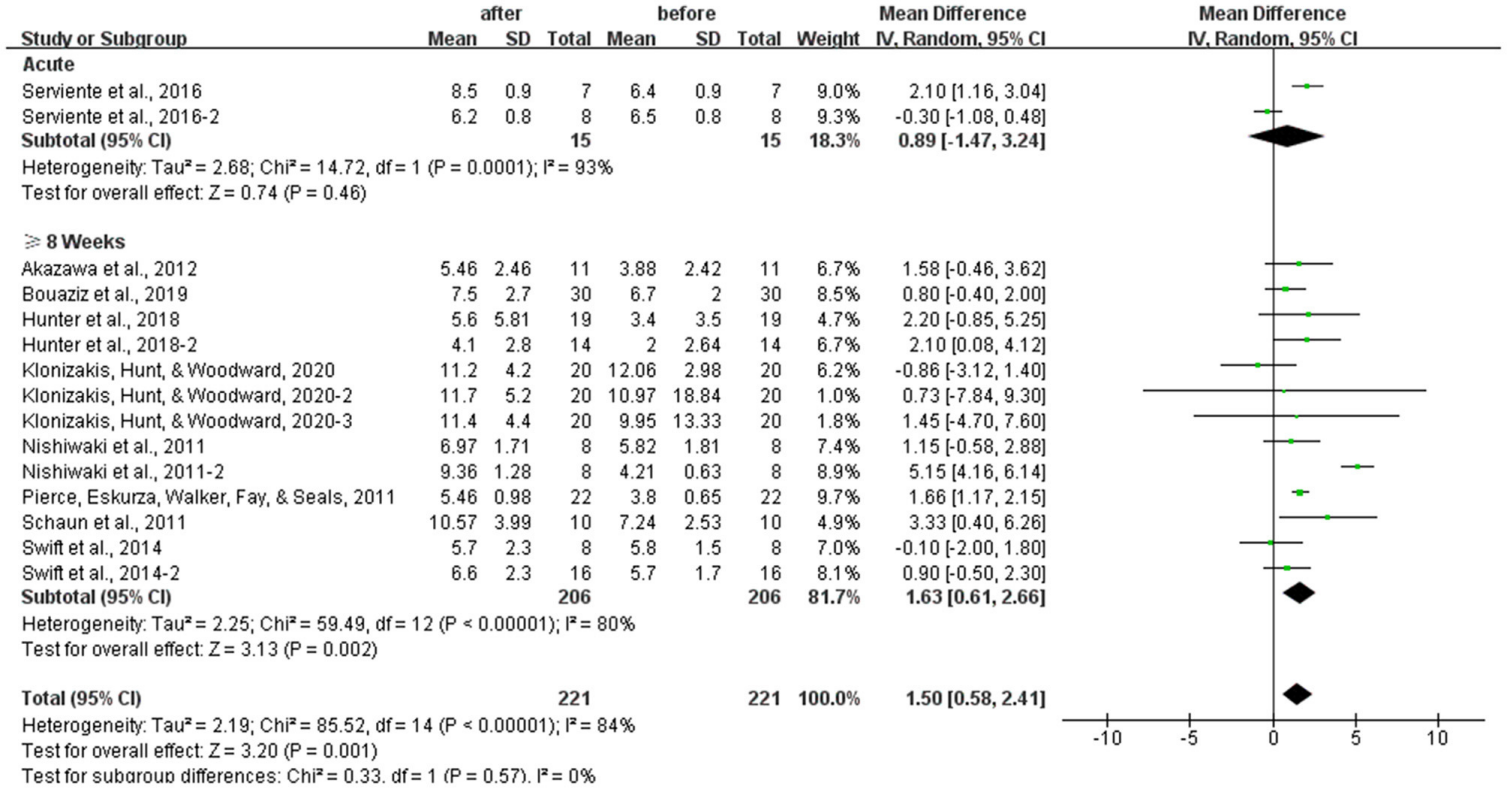
Meta-analysis of the effects of different durations of aerobic exercise on the FMD in healthy middle-aged and elderly people. The pooled estimates were obtained from random effects analysis. Diamonds indicated the effect size of each study summarized as WMD. The size of the shaded squares was proportional to the percentage weight of each study. Horizontal lines represented the 95% CI and the vertical broken line represented the overall effect. One-time acute aerobic exercise had no significant associations with FMD in healthy middle-aged and elderly people [WMD, 0.89 (95% CI, −1.47 to 3.24), *P* = 0.46], while 8 weeks or above of aerobic exercise improved FMD significantly in the middle-aged and elderly people [WMD, 1.63 (95% CI, 0.61–2.66), *P* < 0.05].

The subgroup analysis of different durations of aerobic exercise showed that long-term (8 weeks or above) aerobic exercise increased the FMD significantly [WMD, 1.63 (95% CI, 0.61–2.66], *P* < 0.01], which had a significant heterogeneity (*I*^2^ = 80%, Figure 6). However, one-time acute exercise had no significant associations with FMD in healthy middle-aged and elderly people [WMD, 0.89 (95% CI, −1.47 to 3.24), *P* = 0.46], which had a significant heterogeneity (*I*^2^ = 93%, Figure 6).

### Effects of Different Intensities of 8 Weeks or Above of Aerobic Exercise on the FMD

**Figure 7:**
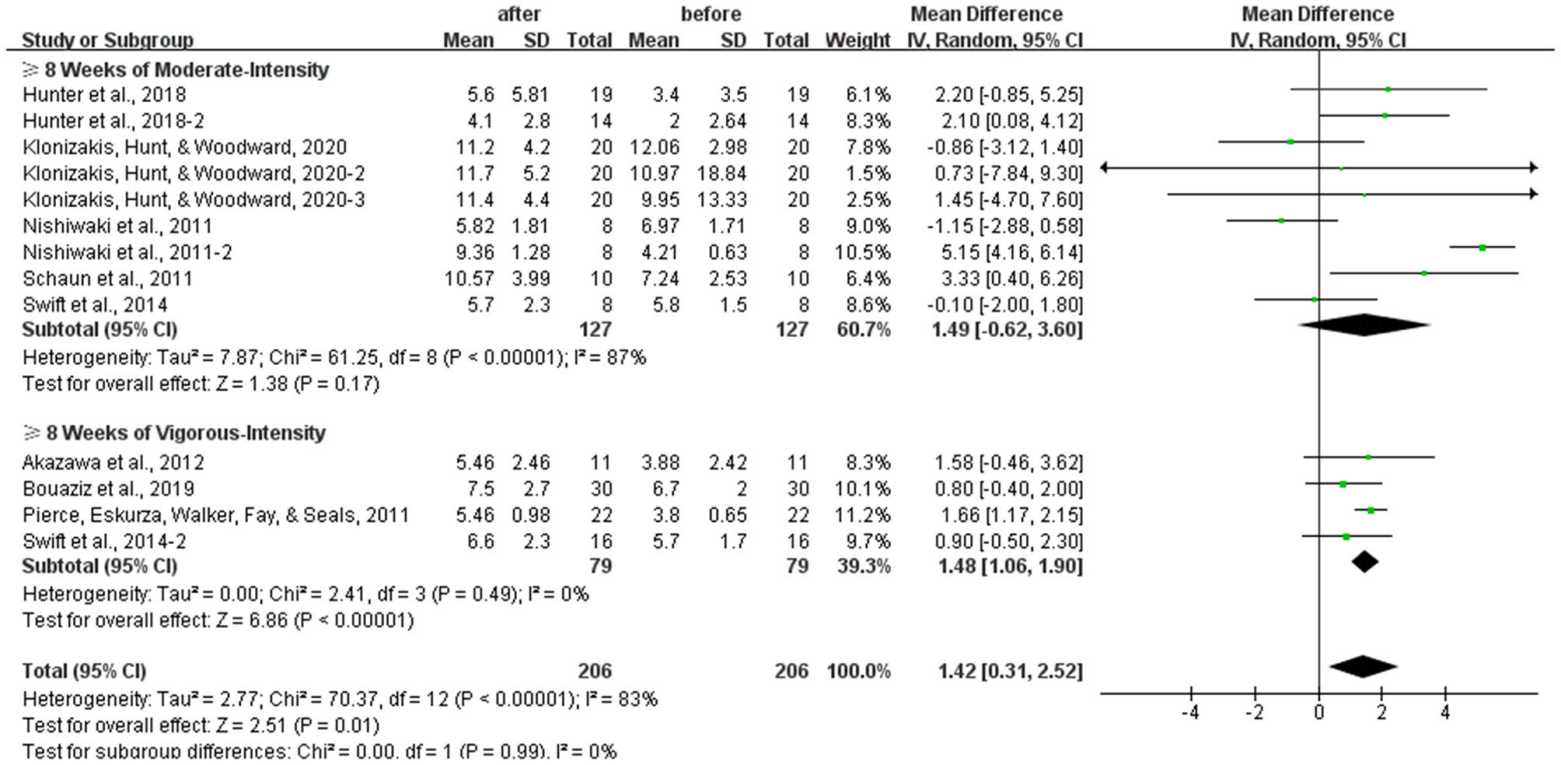
Meta-analysis of the effects of different intensities of 8 weeks or above of aerobic exercise on the FMD in healthy middle-aged and elderly people. The pooled estimates were obtained from random effects analysis. Diamonds indicated the effect size of each study summarized as WMD. The size of the shaded squares was proportional to the percentage weight of each study. Horizontal lines represented the 95% CI and the vertical broken line represented the overall effect. Eight weeks or above of moderate-intensity aerobic exercise had no association with the FMD in healthy middle-aged and elderly people [WMD, 1.49 (95% CI, −0.62 to 3.60), *P* = 0.17], while 8 weeks or above of vigorous-intensity aerobic exercise significantly improved FMD in middle-aged and elderly people [WMD, 1.48 (95% CI, 1.06–1.90), *P* < 0.01].

In the study, we compared the effects of 8 weeks or above of moderate-intensity aerobic exercise and vigorous-intensity aerobic exercise on the FMD, and our results showed that 8 weeks or above of moderate-intensity aerobic exercise had no effect on the FMD in healthy middle-aged and elderly people [WMD, 1.49 (95% CI, −0.62 to 3.60), *P* = 0.17], which had a significant heterogeneity (*I*^2^ = 87%, Figure 7). However, 8 weeks or above of vigorous-intensity aerobic exercise increased the FMD significantly in healthy middle-aged and elderly people [WMD, 1.48 (95% CI, 1.06–1.90), *P* < 0.01}, with no evidence of heterogeneity (*I*^2^ = 0%, Figure 7).

## Discussion

Our meta-analysis showed that aerobic exercise, especially different intensities and durations, significantly improved vascular endothelial function in healthy middle-aged and elderly people, as expressed by increased FMD. Noteworthy, 8 weeks or above of vigorous-intensity aerobic exercise significantly increased FMD in healthy middle-aged and elderly people. However, our meta-analysis did not provide adequate evidence on which exercise intensity was superior in improving vascular endothelial function, since subgroup analysis failed to show significant difference between vigorous-intensity and moderate-intensity. At the same time, it was obvious that aerobic exercise improved vascular endothelial function by increasing the FMD, requiring more proper exercise intensity and longer exercise duration.

Our study showed that aerobic exercise contributed to an overall improvement in the FMD by 1.33%, which was of clinical importance for healthy middle-aged and elderly people. According to previous studies, the increase of the FMD was positively correlated with the reduction of the risk of CVD, which will decrease 13% following every 1% increase in brachial artery FMD (Inaba et al., 2010; Ras et al., 2013; Xu et al., 2014; Matsuzawa et al., 2015; Thijssen et al., 2019). Therefore, we could conclude that people who participated in aerobic exercise for a longer time (8 weeks or above) had better effects on vascular function than those who did not participate in aerobic exercise regularly. The lifestyle of long-term aerobic exercise might provide a protective mechanism for middle-aged and elderly people to slow down the rate of vascular degeneration. Although the mechanism of aerobic exercise improving vascular endothelial function had not been fully revealed, it was speculated that the beneficial effect of exercise on endothelial function might be strengthened through the following mechanisms.

Firstly, there might be a dose-response relationship between vascular endothelial function and aerobic exercise intensity and duration in healthy middle-aged and elderly people. Our results showed that vigorous-intensity exercise increased the FMD significantly, while moderate-intensity exercise had no significant association with FMD in healthy middle-aged and elderly people, which suggested that higher exercise intensity was more effective in improving FMD. Aerobic exercise could produce an increase in blood flow, thus increasing the shear stress on the endothelium to increase the synthesis and bioavailability of nitric oxide (NO) (Simmons et al., 2011; Reynolds et al., 2021), as different intensities of aerobic exercise could produce different arterial pressure, blood flow, and shear stress patterns (Green and Smith, 2018), the improvements of vascular function and vascular remodeling were more obvious in higher intensity aerobic exercise (Tinken et al., 2010; Birk et al., 2012). Ramos et al. (2015) showed that higher intensity aerobic exercise had a greater effect on the endothelial function in adults than moderate-intensity continuous aerobic exercise, which was consistent with our study. In addition, Rakobowchuk et al. (2008) observed the FMD was significantly improved after 6 weeks of 65% VO_2peak_ (vigorous) intensity aerobic exercise, which was also consistent with our study. However, another study reported that both moderate-intensity and vigorous-intensity had a beneficial effect on improving vascular endothelial function (Islam et al., 2021). Therefore, further prospective and intervention studies were needed.

Furthermore, exercise intensity could not explain the improved vascular endothelial function alone, as the acute aerobic exercise of both moderate-intensity and vigorous-intensity did not affect improving the FMD. Harris et al. (2008) found that acute low-intensity (25% VO_2max_), moderate-intensity (50% VO_2max_), and vigorous-intensity (75% VO_2max_) did not influence the FMD response, which suggested that the dose-response relationship of aerobic exercise and vascular endothelial function was dependent not only on the intensity of exercise but also on the duration of exercise.

Secondly, aerobic exercise could reduce the expression of oxidative stress and pro-inflammatory molecules (Teixeira-Lemos et al., 2011). It was reported that regular aerobic exercise could enhance men's vascular endothelial function by reducing oxidative stress and maintaining the bioavailability of NO, which were the factors of endothelial dysfunction (Yoo et al., 2017; Seals et al., 2019). Aerobic exercise could help to restore the function of endothelial progenitor cells, promote endothelial repair, and then promote angiogenesis (Koutroumpi et al., 2012). However, these reactions required a certain exercise intensity and duration, which provided a new idea for increasing the FMD through aerobic exercise to improve vascular endothelial function and prevent CVD. Our results showed that 8 weeks or above of vigorous-intensity aerobic exercise improved the FMD significantly, which suggested that exercise duration was an important factor in improving vascular endothelial function in middle-aged and elderly people. However, this improvement was not observed in acute vigorous-intensity aerobic, and it might be caused by the following aspects. On the one hand, age-related endothelial damage could be prevented by long-time aerobic exercise through improving vascular endothelial function. And the mechanisms included changes in blood flow conditions, increases in blood flow, blood flow speed, and shear stress, and reduces in reactive oxygen species (ROS) production (DeSouza et al., 2000; Taddei et al., 2000), thereby increasing the bioavailability of NO in the vascular endothelium, maintaining vascular homeostasis (Simmons et al., 2011; Reynolds et al., 2021), delaying the rate of vascular degeneration (DeSouza et al., 2000), and finally having an effect on the prevention of age-induced endothelial dysfunction. On the other hand, the role of NO in the endothelium was unlikely to produce a response in a single skeletal muscle exercise (Gilligan et al., 1994), which was consistent with our results.

Third, according to previous studies, the FMD returned to normal after 60 min of acute exercise in elderly men, while postmenopausal women were not affected by vigorous-intensity exercise (Yoo et al., 2017; Seals et al., 2019), which suggested that sex affected the response of the FMD to aerobic exercise in middle-aged and elderly people. Reviewing our study, the heterogeneities in the subgroup might be related to the impaired vascular endothelial function in women perimenopausal or postmenopausal, since the impaired vascular endothelial function was more pronounced in perimenopausal women, which was more common in postmenopausal women with estrogen deficiency (Moreau et al., 2012). Previous study showed that compared with postmenopausal women, only perimenopausal women had improved vascular endothelial function under acute aerobic exercise (Serviente et al., 2016), which might be due to severe vascular endothelial damage caused by estrogen deficiency in postmenopausal women. Moreau et al. (2013) found that 12 weeks of moderate-intensity aerobic exercise could improve the FMD of postmenopausal women who received estradiol hormone therapy during exercise intervention, while FMD improvement was not seen in the postmenopausal women who did not receive estradiol hormone therapy during exercise intervention. The result of moderate-intensity aerobic exercise could not improve vascular endothelial function in postmenopausal women with estrogen deficiency was contrary to a previous study. Santos-Parker et al. (2017) reported that moderate-intensity aerobic exercise improved vascular endothelial function significantly in healthy middle-aged and elderly men, which suggested that estrogen had a protective effect on vascular endothelial function (Taddei et al., 1996). Estrogen could improve the damaged vascular endothelial function by increasing the bioavailability of NO, reducing endothelin-1 (ET-1), and generating vasodilatation to promote endothelial healing and increase angiogenesis (Mendelsohn and Karas, 2005; Chakrabarti et al., 2008). Furthermore, previous studies showed that despite the impaired vascular endothelial function, aerobic exercise could still improve the FMD significantly (Black et al., 2009; Green and Smith, 2018). However, at least 65%-80% HR_max_ and longer exercise duration were required (Moreau et al., 2012), which was also consistent with our results. Therefore, we believed that it was necessary to strengthen exercise stimulation (intensity, duration, or the volume of aerobic exercise) to continuously improve the vascular endothelial function of middle-aged and elderly women, which was consistent with the opinion of Seals et al. (2019).

This study comes with a few limitations that should be taken into consideration. In the process of literature quality evaluation, subjective factors might cause a certain deviation, and there might be some differences in the FMD measurement methods as it was reported that peak expansion measurement cuff release might lead to an underestimation of the real FMD by up to 40% after 60 s (Black et al., 2008), even though this factor seemed to have little impact on the results of the FMD, and these methods were generally well-defined. In addition, only one study on acute exercise was included in the meta-anlysis, it might cause bias to the comparison of exercise duration.

## Conclusions

In summary, this meta-analysis indicated that aerobic exercise, especially 8 weeks or above of vigorous-intensity aerobic exercise, improved the endothelial function in healthy middle-aged and elderly people.

## Data Availability Statement

The original contributions presented in the study are included in the article/supplementary material, further inquiries can be directed to the corresponding author.

## Author Contributions

QY and LY contributed to literature search, figures, study design, data analysis, data interpretation, and writing. GL and HH contributed to data interpretation and writing. YL contributed to study design, figures, data analysis, data interpretation, and writing. All authors contributed to the article and approved the submitted version.

## Funding

LY was supported by Chinese Universities Scientific Fund (2021QN001). GL was supported by Graduate Students' Innovative Scientific Research Program of Beijing Sport University (20212011).

## Conflict of Interest

The authors declare that the research was conducted in the absence of any commercial or financial relationships that could be construed as a potential conflict of interest.

## Publisher's Note

All claims expressed in this article are solely those of the authors and do not necessarily represent those of their affiliated organizations, or those of the publisher, the editors and the reviewers. Any product that may be evaluated in this article, or claim that may be made by its manufacturer, is not guaranteed or endorsed by the publisher.
